# Human organoids in basic research and clinical applications

**DOI:** 10.1038/s41392-022-01024-9

**Published:** 2022-05-24

**Authors:** Xiao-Yan Tang, Shanshan Wu, Da Wang, Chu Chu, Yuan Hong, Mengdan Tao, Hao Hu, Min Xu, Xing Guo, Yan Liu

**Affiliations:** 1 Institute for Stem Cell and Neural Regeneration, School of Pharmacy; State Key Laboratory of Reproductive Medicine; Key Laboratory of Targeted Intervention of Cardiovascular Disease, Collaborative Innovation Center for Cardiovascular Disease Translational Medicine; Nanjing Medical University, Nanjing, China; 2 Department of Neurobiology, School of Basic Medical Sciences; Nanjing Medical University, Nanjing, China

**Keywords:** Stem-cell differentiation, Regeneration

## Abstract

Organoids are three-dimensional (3D) miniature structures cultured in vitro produced from either human pluripotent stem cells (hPSCs) or adult stem cells (AdSCs) derived from healthy individuals or patients that recapitulate the cellular heterogeneity, structure, and functions of human organs. The advent of human 3D organoid systems is now possible to allow remarkably detailed observation of stem cell morphogens, maintenance and differentiation resemble primary tissues, enhancing the potential to study both human physiology and developmental stage. As they are similar to their original organs and carry human genetic information, organoids derived from patient hold great promise for biomedical research and preclinical drug testing and is currently used for personalized, regenerative medicine, gene repair and transplantation therapy. In recent decades, researchers have succeeded in generating various types of organoids mimicking in vivo organs. Herein, we provide an update on current in vitro differentiation technologies of brain, retinal, kidney, liver, lung, gastrointestinal, cardiac, vascularized and multi-lineage organoids, discuss the differences between PSC- and AdSC-derived organoids, summarize the potential applications of stem cell-derived organoids systems in the laboratory and clinic, and outline the current challenges for the application of organoids, which would deepen the understanding of mechanisms of human development and enhance further utility of organoids in basic research and clinical studies.

## Introduction

Animal models and two-dimensional (2D) cell lines have been successfully applied in biomedical fields over the past 100 years for purposes such as improving our knowledge of cellular signaling pathways, developing guidelines for the design of candidate compounds, identifying potential drug targets, and clarifying the underlying pathological mechanisms of diseases. The value of the model systems is evidenced by the widespread use of these systems worldwide in biomedical research today. However, species differences between animals and humans have become major obstacles for the application of animal models in drug discoveries. The main disadvantage of 2D cell lines is their lack of hierarchical structure, dimensionality, cellular diversity, and cell–cell or cell–matrix interactions, which prevents the cell lines from mimicking the cellular functions present in tissues. Tissue explants may transiently capture physiologically relevant cell organization and interactions, but they are difficult to maintain for long periods of time in vitro due to rapid phenotype disappearance.^1^ Learning human genetic diversity and figuring out its impact on disease pathogeneses and drug responses is vital for the achievement of personalized medicine. Hence, it is important to optimize biological systems in order to eliminate the gap between the cellular and organ levels that is present in most current model systems.

The advent of human pluripotent stem cell (PSC) technology and adult stem cell (AdSC; also known as tissue stem cell) culture systems has opened an avenue for the establishment of in vitro human-specific models for the study of genetic variants associated with human disease and the generation of cellular materials on large-scale compound screening.

The first human pluripotent stem cell line, a line of human embryonic stem cells (ESCs), was established in 1998,^2^ almost two decades after the exploration of mouse pluripotent cell lines reported in 1981.^3,4^ Thereafter, Kazutoshi Takahashi and Shinya Yamanaka reprogrammed mouse fibroblast cells to become PSCs in 2006 by inserting four transcription factors encoding genes Oct4, Sox2, Klf4, and c-Myc. These cells, which were similar to ESCs with regard to their gene expression and potential to develop into three germ layers, were termed induced PSCs (iPSCs). Human iPSC (hiPSC) technology was first established in 2007^5,6^ and has been widely used to generate human “disease-in-a-dish” models. This technology may enable personalized disease modeling that will become an important part of precision medicine. Furthermore, the development of clustered regulatory interspaced short palindromic repeat (CRISPR)/Cas9 endonuclease^7–9^ enabled the creation of genetically edited hiPSC-based disease models. Early approaches focused on iPSC-derived 2D cell cultures, which often lack cell–cell interactions, diversity and microcircuits. Recently, the emergence of in vitro human three-dimensional (3D) organ culture approaches has received widespread attention because it allows more complex organ-like structures and different cell types to be modeled simultaneously.

3D organoids derived from primary tissues, ESCs or iPSCs can self-renew, and self-organize, and exhibit multicellularity and functionality similar to those of in vivo organs. The “organoid” was first reported in oncology as a synonym of “teratoma”, as first reported in 1946.^10^ In the 1960s, “organoids” were used to describe organogenesis by cell dissociation and reaggregation experiments performed by developmental biologists.^11^ Previous studies have harnessed the ability of 3D self-organizing from primary tissues, and improvements in organoid systems in the stem cell field were triggered by the establishment of an intestinal organoid culture system in 2009.^12^ Unlike previous systems, these new methods provided a stable system that could sustain the long-term culture of epithelial-like cells from sorted Lgr5^+^ stem cells or dissected crypts by providing in vivo intestinal stem cell niche components, including epidermal growth factor (EGF), Noggin, and R-spondin-1, and embedded in Matrigel to promote expansion.^12^ Notably, these organoids largely reproduced the tissue architecture in vivo and contained a relatively intact complement of stem cells, progenitor cells, and terminally differentiated cell types. By modifying the combination of growth factors and cell isolation procedures, researchers can rapidly alter the 3D culture system to generate multiple kinds of human normal and cancerous organoids, such as brain,^13,14^ colon,^15^ stomach,^16^ and liver organoids.^17,18^

In this review, we discuss recent advances in organoid technologies and describe the main classes of organoids. We critically evaluate their potential applications value in basic biology, disease modeling, therapeutic screening, regenerative medicine, and others. We also highlight existing limitations or challenges that remain to be overcome.

## Organoids from PSCs and AdSCs

**Fig. 1:**
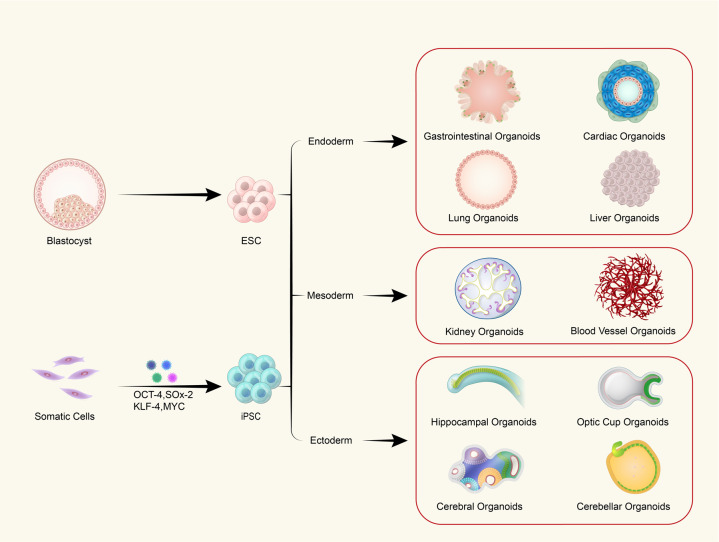
Schematic of the different organoids that can be derived from PSCs. Schematic depiction of different approaches for organoid generation. Embryonic stem cells (ESCs) and induced pluripotent stem cells (iPSCs) are explored for research use. Somatic cells can be reprogrammed into iPSCs by introducing four transcription factors. As development proceeds, blastocysts differentiate into ESCs and further form all three germ layers: the endoderm, mesoderm, and ectoderm. The embryonic endoderm then generates the gastrointestinal system, heart, lungs, and liver. The mesoderm develops into the kidneys and blood vascular system, while the ectoderm forms the neural ectoderm, which eventually becomes the brain with its different regions, including the hippocampus, optic cup, cerebellum, and others. 3D organoid technology can recapitulate all the aforementioned structural and functional features in vitro

**Fig. 2:**
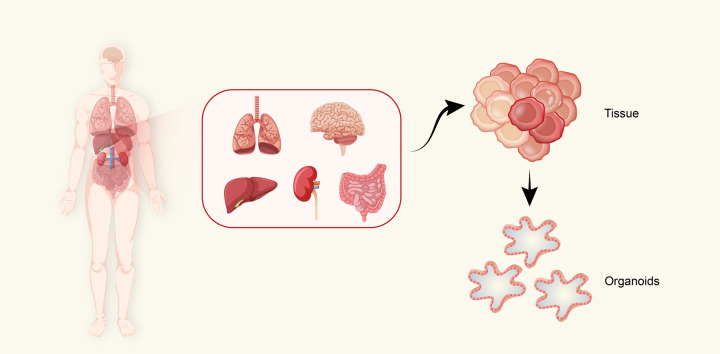
Derivation of organoids from organs. Schematic depicting the stem cell (SC)-derived organoids that could be generated from organs. Unlike embryonic stem cells (ESCs), adult stem cells (AdSCs) are undifferentiated cells located in adult organs, such as the lungs, brain, liver, intestines, and kidneys. After treatment with the appropriate culture conditions, AdSCs are able to form organoids in vitro

Both PSC- and AdSC-derived organoids can form 3D organ-like aggregates to recapitulate the in vivo architecture and function of organs, to some extent. However, there are still some differences between organs and organoids. Zhang et al. firstly generated hPSC-derived spheroids to induce the differentiation of neural lineages and reconstruct neural tube structures in vitro.^19,20^ PSC-derived brain organoid-like spheroids were first reported in 2008 by Sasai’s group and then other organoids were generated from PSCs^21–25^ (Fig. 1). To date, many researchers have established different step-by-step schemes of generating organoids from PSCs; however, these protocols generally take several months, and specific cocktails of growth factors need to be added at each step. Patient-derived somatic cells were first reprogrammed into iPSCs, which then expanded and finally differentiated into specific tissue cell types. PSC-derived organoids usually lose their ability to further expand once the cells reach the time point of terminal differentiation. Due to the pluripotency of PSCs, the cellular components in PSC-derived organoids are relatively complex, including mesenchymal, epithelial and even endothelial components.^26,27^ Thus, PSC-derived organoids are more suitable for studying early organogenesis in human developmental biology because their forming process only occur during embryonic development.^28^ Correspondingly, AdSC-derived organoids were first reported in 2009 for the intestine after identifying small intestinal stem cells by Clevers’ group.^12,29^ Compared with PSC-derived organoids, AdSC-derived organoids are directly obtained from regenerative human adult tissues with a simpler procedure in less time^27,30^ (Fig. 2). The restricted potency of AdSCs leads to a single epithelial cell type in all AdSC-derived organoids.^28^ Notably, the maturity of AdSC-derived organoids is closer to that of adult tissue than that of PSC-derived organoids, so AdSC-derived organoids provide a better overview of adult tissue repair and viral infection disease,^27^ except the tissues with stemness that are unably obtained from adult organs, such as brain, heart, and islet. The resulting structures derived from normal or diseased epithelial tissues can be expanded in vitro for a long time while maintaining gene stability, thus making them ideal for the expansion of cells from individual patients and normal groups and facilitating their potential application in the study of new therapeutic strategies. Collectively, the evidence suggests that both PSC-derived organoids and AdSC-derived organoids can be used as complementary tools in future scientific research and potential personalized medicine. To date, biomedical scientists have developed methods to generate a variety of PSC-/AdSC-derived structures/tissues “in a dish”.

## Brain organoids

The central nervous system (CNS) develops from the neural tube, a structure that is formed by the folding and fusion of neural plates. The morphogenetic steps/patterning are coordinated by morphogen gradients along the dorsal–ventral (D–V) axis and anterior–posterior (A–P) caudal axis. The gradient of morphogens defines the identity of neural progenitors in a specified domain. Together with regional localization of intrinsic factors, this can promote cell commitment toward specific lineages to form multiple brain regions, each containing a cytoarchitecture necessary for its particular function. Protocols have recently been established for in vitro 3D cell cultures of specific areas of brain neural tissue, which has made it possible to investigate early developmental events and disease mechanisms of human brain.

### Cerebral organoids

**Fig. 3:**
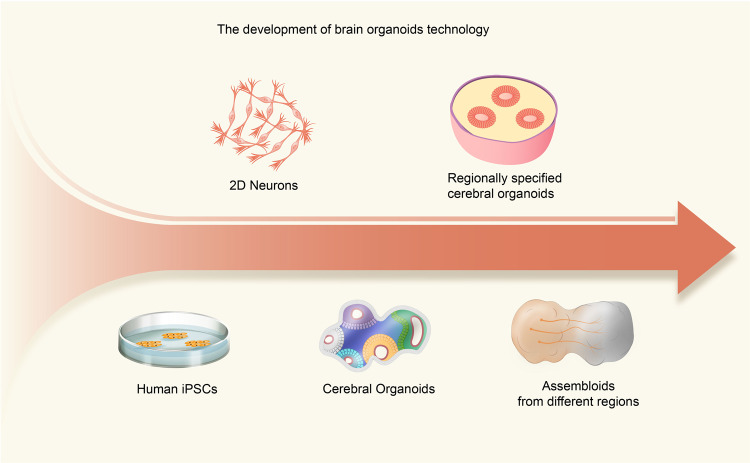
Graphical summary of major milestones in the development of brain organoid technologies. The advent of human iPSC (hiPSC) technologies has enabled the production of specialized human cells in large quantities for disease modeling. hiPSCs have been widely available for 2D culture systems, but 3D models are regarded as more advanced alternatives to mimic human brain complexity. Lancaster et al. produced cerebral organoids by embedding embryoid bodies into an extracellular matrix (ECM), giving hiPSCs the most freedom for self-organization; the resulting organoids contained diverse tissues, including the retina, forebrain, midbrain, hindbrain, and choroid plexus. However, at the early stage of brain region-specific organoid culture, patterning factors were used to determine the fate of progenitor cells and then removed at the further differentiation stages. These guided protocols allowed the generation of two or more organoids resembling various brain regions that can then be used to fuse to form assembloids, which can be used to simulate the interactions between different brain regions

It is well known that the brain is a complex organ of the human body, of which the cortex occupies the largest volume. The development of the human cerebral cortex originates from the rostral neuropore of the neural tube. At present, exploration of human brain development and the pathogeneses of neurological diseases is mostly realized with model animals. However, the human brain is different from the brains of other species, with more sophisticated structures and higher cognitive and mood functions. Therefore, the emergence of 3D brain organoid cultivation technology has provided a new option to explore the formation and functional network of the human brain. Here, we summarize the development of human cerebral cortex organoid systems (Fig. 3).

**Fig. 4:**
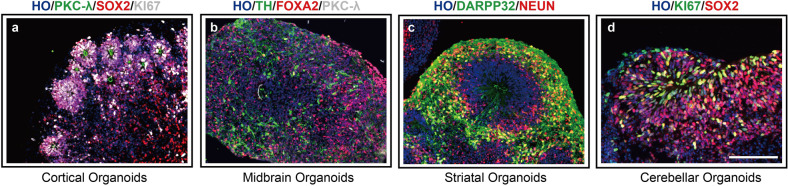
“Mini-brains” generated from PSCs. “Mini-brains” representative of different brain region identities were generated in Yan Liu’s laboratory. **a** Immunofluorescence image of cortical organoids grown from pluripotent stem cells (PSCs) with multiple rosette-like structures. Shown are immunohistochemical staining images for the adherens junction marker PKCλ (green), the progenitor marker SOX2 (red) and the proliferative marker KI67 (green) after 30 days of differentiation. **b** Immunofluorescence of the midbrain dopamine (DA) neuron marker TH (green), the floor plate progenitor marker FOXA2 (red) and PKCλ (green) at 50 days in midbrain organoids. **c** Image of striatal organoids stained for the medium spiny neuron (MSN) marker DARPP32 (green) and the mature neuron marker NEUN (green). **d** Staining for KI67 (green) and SOX2 (red) in cerebellar organoids at 30 days. Scale bar, 100 μm

In 2008, Eiraku et al. cultured embryoid bodies with specific growth factors in a serum-free floating culture of embryoid body (EB)-like aggregates with quick aggregation (SFEBq) system, which formed cortical-like structures with apical–basal polarity and generated cortical progenitors and functional neurons.^21^ Based on the SFEBq technique, Lancaster et al. embedded embryoid bodies in Matrigel and added growth factors for neural development during suspension culture.^13^ After long-term neural differentiation culture, more complex cell composition and structure are obtained. This was the first time that the concept of 3D cerebral organoid using human PSCs was demonstrated. To better represent the structural level of the brain, Sasai’s team^31^ selectively induced PSCs to differentiate into neuroepithelial cells by inhibiting both WNT and transforming growth factor-β (TGF-β) and produced cortical organoids with a relatively complex cortical morphology. Qian et al.^32^ optimized the differentiation method by adding BDNF, GDNF, TGF-β, and cAMP, which generated cortical organoids mimicking the six-layer structure of cortical tissue. Currently, constructed cortical organoids mainly contain neural progenitor cells, intermediate neural progenitor cells, γ-aminobutyric acid (GABA) neurons, glutamatergic neurons, and glial cells that form functional neural networks, thus demonstrating that cortical organoids can simulate human cortex development and function.^33^ Recently, our group applied single-cell sequencing to compare the gene expression profiles of single cells in cerebral organoids to the profiles during fetal human developmental stages. Within 30 days, the cerebral organoids exhibited the greatest correlation with transcriptomes of post-conception week (PCW)-8 and PCW-9 samples, emphasizing that human early cortical developmental stages can be studied with organoid cultures^34^ (Fig. 4).

In recent years, as cerebral organoid technologies have become more sophisticated, an increasing number of new technologies, such as assembly^35^ and vascularization technologies,^36^ have been developed. In summary, cerebral cortex organoids could be applied to solve current challenges in the study of brain development and neurological diseases.

### Hippocampus

The hippocampus is an S-shaped structure located in the medial part of the temporal lobe containing two parts: the cornu ammonis (CA) and dentate gyrus (DG).^37^ The hippocampus develops from the dorsomedial telencephalon and plays a vital role for learning and memory.

By optimizing the cortical organoid protocol, Sakaguchi et al. used hPSCs to generate 3D self-organizing hippocampal tissue that contained choroid plexus- and medial pallium-like structures.^38^ The protocol mainly involved inhibition of SMAD signaling to generate a telencephalon and reactivation of the WNT and bone morphogenic protein (BMP) pathways to induce dorsalization. After long-term culture, PSCs were successfully induced to form hippocampal organoids with the generation of hippocampal DG- and CA-type neurons, which promoted the formation of functional neuronal networks with synaptic connections.^38^

Many patients with mental diseases, such as Alzheimer’s disease and schizophrenia, suffer from hippocampal damage and declines in learning and memory.^39^ Hippocampal organoids offer new tools for studying such diseases. However, reconstruction of the complex hippocampal neural circuit in vitro remains a challenge.

### Striatum

The striatum develops from the lateral ganglionic eminence (LGE) during mid-gestation, linking many neuronal circuits in the human brain, and the striatum malfunction is involved in many diseases, such as Huntington’s disease^40^ and addiction.^41^ In 2020, Miura et al. first generated human striatal organoids containing morphologically and functionally mature striatal neurons called human striatal spheroids (hStrSs).^42^ Activation of retinoid receptors by SR11237 combined with IWP2 and activin A treatment significantly increased the number of Genetic-Screened Homeobox 2 (GSX2)-positive and COUP-TF-interacting protein 2 (CTIP2)-positive cells. Furthermore, they assembled hStrSs with cortical organoids to model human corticostriatal circuits in vitro and found that the intrinsic functional maturation of striatal neurons was accelerated for the assembled hStrSs compared with non-assembled hStrSs. To highlight practical applications, they used this system to study the molecular mechanism of cellular defects after the loss of SHANK3 in Phelan–McDermid syndrome. In the future, striatal organoids could be useful for modeling the pathology of many other neuropsychiatric disorders, such as addiction, autism, obsessive-compulsive disorder, Tourette syndrome, and Huntington’s disease.^43,44^

### Midbrain

The midbrain, developed from the mesencephalon, is the forward-most part of the brainstem and consists of tectum and tegmentum. The midbrain plays important roles in sleep, motor control, arousal (alertness), wakefulness, and temperature regulation.

In 2016, Jo et al. first established human midbrain organoid expressing markers of midbrain dopaminergic neurons such as FOXA2 and TH.^45^ Besides neuromelanin-like granules which are similar to human substantia nigra were also recapitulated in their model. Thereafter, Qian et al. reported another protocol for generating midbrain organoid.^32^

To date, two main canonical protocols have been developed. The first is the EB-based protocol. The initial step of this method is EB formation via floating culture and the addition of SB, which inhibits the Activin/TGF-β signaling pathway, combined with the BMP inhibitor DMH1/Noggin/LDN. In addition, CHIR, normally used as an inhibitor of GSJ-3β, was added to activate WNT signaling and induce midbrain/hindbrain identity.

Furthermore, activation of the SHH signaling pathway in midbrain organoids is essential, since this pathway plays the most important role in the patterning of floor plate precursors. SHH or smoothened agonist (SAG) was the first option as the molecular activator. Under treatment with these molecules, up to 60% TH-positive cells could occur in the midbrain organoids.^45,46^

Another canonical protocol is the floor-plate (FP)-based protocol. The main difference between the EB and FP protocols is that the FP method does not involve the formation of EBs, which can be passaged and amplified.^47^ Both EB- and FP-derived midbrain organoids showed structural and functional characteristics consistent with those of the in vivo midbrain, including cell composition types, defined spatial organization, transcriptional characteristics, neuromelanin granule expression, intrinsic membrane properties and electrophysiological discharge mode.^48,49^ Notably, the concentrations of these morphogens are vital in midbrain organoid differentiation.^47^ Researchers have proven that midbrain organoids derived from PD patients or through gene editing exhibit the classical pathological phenotypes of PD, for example, a decreased number of DA neurons, reduced neuronal network complexity ^50^ and exhibition of Lewy body-like inclusions.^51,52^ Studies on midbrain organoids have provided insights into the genetics and pathophysiology of PD.

### Cerebellar organoids

Recent studies have shown that the cerebellum is not only a motor control organ,^53^ but also contributes to emotion, language, and cognition.^54–56^ Developmental deficits and/or impairment of the cerebellum can result in motor-related or cognitive dysfunction, including medulloblastoma and spinocerebellar ataxia.^57–59^ The cerebellum is a derivate of the anteriormost part of the hindbrain (rhombomere 1). After the formation of the isthmic organizer,^60^ the development of the cerebellum starts.^61^

Therefore, Yoshiki Sasai’s group focused on initiating an endogenous self-inducing isthmic formation process to produce functional Purkinje cells. They added insulin and FGF2, which work as weak caudalizing signals, at the early stage of SFEBq culture. To achieve dorsal specification, cyclopamine was added to the system beginning on day 7. Eventually, based on these conditions, the in vivo microenvironment of cerebellar development was successfully recapitulated. Thus, functional Purkinje cells were achieved.^62^ On the basis of this program, Muguruma et al. optimized culture conditions for the differences between mouse ESCs (mESCs) and hPSCs.^63^ V-bottomed 96-well plates were used to promote human ESC^64^ reaggregation. SB431542, a TGF-β receptor blocker, was added to the system during the first two weeks to inhibit the differentiation of mesenchymal cell and promote the differentiation of neuroectodermal. Markedly, the researchers also found that the addition of FGF19 and SDF1 could promote self-organization of the polarized cerebellar plate neuroepithelium (CPNE) structure. The cerebellar organoids generated under these conditions could model the first trimester of the human cerebellum. Nevertheless, further maturation of neurons in embryonic cerebellar organoids into cerebellar neurons has remained a challenge, and coculture with granule cells of different origins has generally been applied to generate functional cerebellar neurons.^63,65,66^ More recently, size control for cell aggregation and spinning bioreactors have also been used to induce cerebellar organoids with greater maturity.^67^ Overall, cerebellar organoids contain the major cell types of the cerebellum and offer new insights into the developmental and disease mechanisms related to cerebellum. Using patient-derived iPSCs, cerebellum organoids have also been applied for disease research and drug discovery.

### Thalamic organoids and hypothalamic organoids

The thalamus and hypothalamus originate from the diencephalon. As the thalamus is an information exchange center in the brain, thalamic neurons consist of distinct nuclei and project to the cerebral cortex and other subpallial regions in an ordered manner. The hypothalamus is associated with many physiological functions, including circadian rhythm, thermoregulation, and hormone secretion.^68^

Suzuki-Hirano et al. reported that BMP7 could be applied to maintain thalamic identity.^69^ In addition, treatment with insulin facilitates the caudalization of telencephalon tissues, and MEK-ERK inhibitor treatment suppresses excessive caudalization when thalamic neurons are induced to differentiate from mESCs.^70^ Xiang et al. established a protocol that differentiated hPSC-derived specified human brain organoids to mimic the thalamus. They produced a 3D model for recapitulating axonal projections reciprocally between the human cortex and thalamus by fusing cortical and thalamic organoids for the first time.^71^ More recently, Fligor et al. reported the formation of 3D assembloids from cortical, thalamic, and retinal organoids, offering a long-distance projection model to study the physiological process of visual development.^72^ They found that the retinal organoids showed remarkable survival improvement, with the axons of retinal ganglion cells extending into the thalamic organoid and thalamic neurons entering the cortical organoid.

In 2008, Wataya et al. showed that mESCs could differentiate into RAX+, SIX3+ and VAX1+ rostral hypothalamic progenitor-like cells with mature functions.^73^ Suga et al. then generated adenohypophysis aggregates with hypothalamic neuroectoderm from mESCs to mimic the endocrine function of the hypothalamus for the first time.^60^ Subsequently, by adding Wnt, BMP, Notch inhibitors, and SHH agonists into culture systems, researchers successfully induced the differentiation of hypothalamic neurons with neuropeptide secretion.^74,75^ Thereafter, Qian et al. optimized the protocols for hypothalamic organoids to study Zika virus (ZIKV) infection.^32^ Recently, Huang et al. reported a protocol to generate iPSC-derived hypothalamic arcuate organoids that can be used to model Prader-Willi syndrome.^76^ Overall, the technology of guided region-specific organoids is useful for studying thalamus- and hypothalamus-associated physiology and diseases.

## Retinal organoids

The retina is a highly complex laminated structure of the eye whose most important function is to convert light energy into visual perception. During eye development, the optic sulcus, which develops from the neuroectoderm, sinks deep and forms a chamber called the optic vesicle. The latter then moves inward to form a double-layered cup-shaped structure, which is called the optic cup. The optic cup is composed of the outer retinal pigment epithelium and the inner neural retina.

After decades of exploration, scientists have successfully developed retinal organoids that closely resemble the bona fide retina in both structure and function. As early as 1992, Layer et al. reaggregated retinal cells dissociated from chick and mouse tissue to form spheroids.^77^ In 2011, Yoshiki Sasai’s groups derived mammalian retinal tissue from in vitro stem cells, which was considered a milestone in 3D organoid culture in the CNS.^78^ The researchers first generated retinal organoids with autonomous formation of the optic cup structure from mESC aggregates.^78^ They further established an optic cup model from hESCs that was much larger than the mouse-derived optic cup.^79^ In addition to the optic cup-like structure, they also reported the emergence of ciliated limbal like stem cell niches in human retinal tissue.^80^ To achieve such niches, they induced neural retina differentiation via timed BMP4 treatment followed by GSK3 and FGFR inhibition with subsequent removal of this inhibition. This stepwise induction-reversal approach generated retinal pigment epithelium aggregates at the edge of the central peripherally polarized neural retina.

In addition to Sasai, many other researchers have also produced excellent works regarding retinal 3D culture and regeneration in vitro.^81–86^ For example, Lamba et al. directly differentiated cells by using specific neuralizing factors, resulting in the reaggregation of retinal laminated structures.^87^ Scientists have also developed retinal organoids that showed physiological responses to light stimulation.^88,89^

A recent study established a protocol to obtain optic vesicle-containing brain organoids (OVB organoids) from hiPSCs.^64^ OVB organoids were composed of many different developing optic vesicle cell types, including retinal progenitor cells, retinal pigment epithelial cells, lens-like cells, and primitive corneal epithelial. The authors found that the photosensitizing activity of OVB organoids could be triggered by various light intensities. As a result, retinal organoids are promising tools to investigate eye development and visualization function.

## Kidney organoids

The kidneys comprise nephrons developed from the metanephric mesenchyme (MM) as well as collecting ducts and ureters originated from the ureteric bud (UB). Along with a complex structure, the kidneys have more than 25 specialized cell types,^90^ playing a prominent role in removing harmful metabolites and maintaining homeostasis in vivo. Hence, the kidney is a major target of toxins and is highly susceptible to toxicity because of its unique urine-concentrating function and its integration with the complex vascular network.^91^

Based on Taguchi’s protocol, the first method for generating kidney organoids was established in 2014 and involved the use of mESCs and hiPSCs.^22^ Since the UB and MM arise from the anterior intermediate mesoderm (IM) and posterior IM, respectively, Takasato et al. optimized their protocol and induced organoids consisting of patterned segmented nephrons surrounded by endothelial cells and renal interstitium.^92^ As a result, they generated complex hESC-derived multicellular kidney organoids containing UBs and MM synchronously for the first time. In addition, the transcription profiles of the kidney organoids generated in vitro were similar to those of human fetal kidneys.^93,94^ Researchers have also achieved glomerular vascularization and tubular functional maturation to better mimic the structure and function of the kidneys in vivo.^93,95,96^ Notch signaling activation for the differentiation of proximal tubules has been identified using these kidney organoids, suggesting the feasibility of in vitro organs as pharmacological screening platforms.^97^ Additionally, several compounds, including doxorubicin, interleukin-1β, adriamycin, and cisplatin, have been proven to be toxic to the kidney by using kidney organoids.^94,98–100^ Notably, after transplantation, the engrafted kidney organoids became more mature and vascularized in host mice, which demonstrated that kidney organoids may be novel sources for autologous transplantation.^101,102^

## Liver organoids

The liver, a major organ found only in vertebrates, is located in the right upper abdomen, performs many essential biological functions, such as metabolism and detoxification with its remarkable ability to regenerate upon serious damage. The major functional cell types in the liver are endodermal-derived hepatocytes and cholangiocytes. Michalopoulos et al. isolated rat hepatocytes and generated a liver-derived 3D culture system for the first time in 2001.^103^ Although long-term viability was difficult to sustain for later stages of research, the system retained limited structural and functional features. In 2011, Sekiya and Suzuki induced the mouse embryonic and adult fibroblasts to hepatocyte-like cells with two factors: Hnf4α and together with one of Foxa1, Foxa2 or Foxa3.^104^ By overexpressing Gata4, Hnf1α and Foxa3, Huang et al. directly converted adult fibroblasts to functional hepatocyte-like cells.^105^ Huch et al. created liver organoids in 2013 and reported that damage to the mouse liver led to Lgr5+ cell appearance.^106^ They found that even single cells could differentiate into hepatocytes and form functional liver organoids over several months in vitro under treatment with the Wnt agonist RSPO1. To further characterize organogenesis, they induced primary human bile duct cells to form 3D liver organoids and demonstrated that cyclic adenosyl monophosphate (cAMP) and a TGF-β inhibitor play key roles in long-term culture.^17^

The posterior foregut gives rise to the liver bud during early organogenesis. Thus, Takebe et al. first cocultured hiPSC-derived hepatic endoderm cells (hiPSC-HEs), human umbilical vein endothelial cells (HUVECs) and mesenchymal stem cells (MSCs) to mimic liver bud formation and rescued mouse hepatic function after transplantation.^107^ They soon overcame the availability limitation by differentiating liver buds completely from hiPSCs.^108^ To recapitulate liver regeneration after acute injury, Hu et al. reported a method for generating mouse and human hepatocyte organoids with proliferative damage responses for long periods of time.^109^ Similarly, Peng et al. expanded mouse hepatocyte organoids, focusing on the injury-induced inflammatory cytokine TNFα, which promoted the expansion of hepatic cells.^110^ Hepatocytes secrete the bile that is collected by bile canaliculi, but these anatomical structures can rarely be recapitulated. However, Vyas et al. established a model of self-assembled human liver organoids with hepatobiliary organogenesis to some extent.^111^ Prior further identified that Lgr5+ hepatoblasts could differentiate into embryonic liver organoids that give rise to both hepatocyte and cholangiocyte progeny.^112^ Recently, Ramli et al. established a method to generate hepatic organoids containing a functional bile canalicular system and model troglitazone-induced cholestasis in vitro.^113^ In conclusion, the emergence of liver organoid technology will aid in organogenesis modeling, liver transplantation, and drug screening.

## Lung organoids

The lung arises from the developing ventral foregut endoderm.^114^ It is composed of complex conducting tubes that terminate in numerous highly vascularized distal sacs for the purpose of efficient gas exchange between the air and blood. The airways mainly consist of four epithelial cell types: goblet cells, ciliated cells, club cells (also known as Clara cells) and basal cells.^115^ In contrast, the alveolar epithelium covers >99% of the inner surface of the lungs and is mainly lined by two epithelial cell types: alveolar epithelial type I cells (AECIs), which are responsible for gas exchange, and alveolar epithelial type II cells (AECIIs), which mainly secrete surfactants.

To mimic the lung in vitro, Freeman et al. first developed the concept of mouse lung organoids in 1980.^116^ Lung tissue of mouse full-term embryos was cultured with sterile pigskin dermal collagen as a substrate, and various sizes of ductular structures were observed. Although the process was complex and not suitable for human study, it laid the foundation for in vitro lung organoids generation. After three decades, Kotton et al. efficiently obtained purified endodermal precursors from mESCs by inhibiting TGFβ/BMP signaling and then stimulating BMP/FGF signaling.^117^ Upon induction, they demonstrated the directed differentiation of primitive lung progenitors that could reproduce lung development. Their study formed a sound basis for subsequent attempts to generate lung organoids.

Shortly afterward, Rossant and colleagues first described the production of lung organoids from hiPSCs.^23^ After treating the cells with exogenous factors, they used air–liquid interface culture at the last stage of differentiation to obtain mature airway epithelial cells for cystic fibrosis modeling. Snoeck et al.^118^ and Spence et al.^119^ then successfully established two similar step-by-step protocols for the directed differentiation of hPSCs into lung airway and alveolar epithelial cells, which were then expanded into lung organoids. After long-term induction of hPSCs in vitro, not only four types of airway epithelial cells but also functional AECIs and AECIIs were found to exist in both studies. Chen et al. further generated lung bud organoids that contained pulmonary endoderm and mesoderm to illustrate lung development more finely.^120^ In addition, Sachs et al. found that airway organoids derived from metastasis biopsies and lung cancer resections retained the features of tumor cancer gene mutations and histopathology, which is valuable for drug screening.^121^

Lung has an extraordinary ability to regenerate and to restore its function after injury, which makes it possible to overcome the complexity and tedious procedure of differentiation from PSCs and generate lung-like tissues directly from AdSCs.^122^ The airway contains basal stem cells that act as progenitors for self-renewal and secretory club cells that act as progenitors by showing plasticity after injury, while the alveolar epithelium has AECIIs to replenish lost AECIIs and produce AECIs after injury by proliferation.^123–126^ Based on the regenerative capacity of lung cells, many groups have generated organoids from mouse and human airway basal cells,^123,127,128^ airway secretory club cells^124,129,130^ and AECIIs.^125,131^

Due to the possibility of reproducing lung tissue in vitro and their ability to simulate important gas exchange functions, lung organoids have emerged as potential research tools for many respiratory diseases. For instance, many researchers are studying the effect of the recently emerging severe acute respiratory syndrome coronavirus 2 (SARS-CoV-2) on the respiratory system by using lung organoids.^130,132–136^ Thus far, lung organoids have laid a foundation for studying different pandemic-causing viruses and discovering potential therapeutic targets in the respiratory system.

## Gastrointestinal organoids

The primary functions of the gastrointestinal (GI) tract are digestion, absorption, excretion, and protection.^137^ As the most important parts, the stomach and intestine are fundamentally responsible for digestion and absorption. The first intestinal organoid model was developed in 2009^12^ by Toshiro Sato and his colleagues. This model contained Lgr5^+^ AdSCs from mouse intestinal crypts embedded in an extracellular matrix (ECM) and cultured with essential growth factors, including R-spondin-1, EGF and the BMP inhibitor Noggin. One year later, the first gastric organoid culture system was established,^138^ derived from Lgr5^+^ stem cells in antrum glands on the base of the intestinal organoid culture system with additive fibroblast growth factor 10 (FGF10) and the hormone gastrin.

Organoids produced by protocols mentioned above consist only of epithelial cells in stomach or intestine. To obtain cell culture models with more cell types and more germ layers in order to approximate physiological conditions, researchers developed models of intestinal and gastric organoids from PSCs in 2011^24^ and 2014,^139^ respectively. Gastric organoids could also be generated directly from samples of the human gastric corpus.^16,140^ Besides epithelial cells, organoids derived from PSCs contain mesenchymal cells, which is the major difference from AdSCs-derived organoids.^27,141^ Since PSC-derived organoids encompass more cell types, the absence of adjacent cells, such as neural cells and immune cells, is still a limitation of current organoid culture systems. Recently, multiple methods, including coculture,^142^ biomaterial-based methods^143,144^, and 3D bioprinting,^145^ have been utilized to optimize the culture systems of gastric and intestinal organoids.

In the human body, both the stomach and intestines are closely associated with the microbiota. The microbes in the microbiota usually reside on the mucosal surface of the gastric or the intestinal lumen^146^ and have a great impact on the GI biology of the host; for example, they can affect immune homeostasis and participate in competitive inhibition.^147,148^ In particular, increasing amounts of evidence have indicated that the microbiota in intestine greatly affects drug pharmacokinetics and therapeutic effects, the importance of which has become increasingly known through recent drug development and clinical trials.^149^ Intestinal commensal bacteria such as *Lactobacillus*, when cocultured with intestinal organoids, could promote organoid proliferation and differentiation into Paneth cells.^150^ Moreover, these bacteria could protect organoids from damage caused by inflammatory factors, such as TNF-α.^151^

Studies on coculture with pathogenic *Escherichia coli*,^152,153^ *Cryptosporidium*,^154^ *Salmonella*^155^, and *Clostridium difficile*^156^ bacteria have also been conducted to shed light on the effects of bacterial pathogens on the intestinal epithelium. Many severe gastric diseases, such as peptic ulcer disease and gastric cancer, are closely related to *Helicobacter pylori*.^157^ To investigate the pathogenesis of *H. pylori* infection and screen effective drugs, McCracken and his colleagues injected *H. pylori* into gastric organoids derived from PSCs and found a mechanism in which GagA bound to the c-Met receptor of epithelial cells, leading to overexpression of epithelial cells.^139^ Wroblewski et al. showed that CagA and β-catenin signaling regulated the proliferation of gastric organoids, inducing incorrect localization and suppression of the tight junction protein claudin-7 related to cancer.^158^

## Cardiac organoids

Heart is the first functional organ during human embryonic development, originates from the splanchnic mesoderm and develops into the cardiac crescent. Through looping of the original primary heart tube, the four individual heart chambers (right atrium, RA; left atrium, LA; right ventricle, RV and left ventricle, LV) are gradually formed. The endocard and the epimyocard, two different layers of the early heart, eventually develop into endocardium as well as myocardium and epicardium.^159,160^ Signals from the adjacent endoderm and overlying ectoderm together constitute a unique signaling environment that drives cardiac differentiation and includes BMP, FGF, and WNT signals.^161^ Mutations in the corresponding signaling pathways and transcription factors during the early embryogenesis often cause heart defects.^160,162,163^

Artificial heart cardiomyocyte tissue has been successfully constructed with bioengineering approaches including geometric confinement, molds and poles. The laboratory of Christine L. Mummery has established mouse and human PSC-derived multi-cell-type 3D cardiac microtissues composed of cardiomyocytes (CMs), cardiac endothelial cells (ECs), and cardiac fibroblasts, the three main cell types of the heart.^25,164^ Insights into the specification of two cardiac origins, the FHF and the second heart field,^165^ have been provided by studies on precardiac organoids.^166^

The first description of the generation of mESC-derived cardiac organoids with atrium- and ventricle-like 3D structures was reported by the laboratory of Fumitoshi Ishino.^167^ The use of ECM supplied with the laminin-entactin (LN/ET) complex and exogenous fibroblast growth factor 4 (FGF4) under serum-free conditions allowed the production of 3D heart organoids containing CMs, ECs, smooth muscle cells (SMs) and potential cardiac autonomic neurons.^167^ Recently, Drakhlis et al. generated hPSC-derived 3D heart-forming organoids (HFOs) through the application of Matrigel and biphasic WNT pathway modulation.^168^ The HFOs resembled the early cardiac and foregut anlagen. Almost at the same time, hPSC-derived self-organizing cardiac organoids, cardioids, were established by Sasha Mendjan and coworkers. Different from HFOs, cardioids not only contain different cell types involved in cardiac development but also have atria and ventricle-like chamber structures.^169^ Rapid and complex development processes are now feasible due to the generation of these cardiac organoids. Researchers have also used these cardiac organoids to study congenital cardiac malformations and developmental injury via conditional gene knockout or cryoinjury.

## Vascularized organoids

Organ needs blood vessels to ensure sufficient nutrient and oxygen supply. Therefore, creating a suitable and reliable model with a vascular system in vitro is vital for the development of biomedicine. Wimmer et al. induced hPSCs to differentiate into mesoderm and formed 3D human blood vessel organoids after induction with the growth factors VEGF-A and FGF2.^170^ After transplanting to mice, the blood vessel organoids developed into a complete vascular system including arteries and capillaries. Blood vessel organoids could be used to identify the pathways involved in diabetic vascular changes and play a key role in developing drugs to alleviate diabetic microvascular changes.

Organoid vascularization is important for recapitulating normal human physiological conditions and for long-term organoid culture. Vascularization of cerebral organoids was considered as a key step in the study of neurological diseases. Pham et al. cocultured endothelial cells with cerebral organoids and generated endogenously vascularized whole-brain organoids.^36^ It has been verified that the vascularization of organoids increased their survival rates in vitro. Other strategy was reported to engineer hESCs to express human ETS variant 2 (ETV2) ectopically in human cortical organoids (hCOs) to form vascular-like networks in the hCOs.^171^ These vhCOs gained several blood–brain barrier (BBB) characteristics that resembled the vasculature in the early prenatal brain.^171^ The researchers found that the vhCOs could form a functional vascular-like network exhibiting blood-barrier characteristics, which resulted in improved maturation of cortical organoids.

In addition to the vascular brain organoids, other organoids such as liver,^107^ skin^172^, and kidney organoids^93^ could be vascularized, facilitating the development of physiological and pathological models relevant for basic and clinical research and for further regenerative medicine and pharmacology research.

## Multi-lineage organoids

It is well known that cells in the human body do not function in monolineage isolation. The connections among different cell types are of vital importance for physiological and pathological studies.^173^ Although the established organoids already contain many cell types, it is still difficult to mimic the interactions in real tissues due to the lack of other lineages of cells from different organs.^174^ Therefore, a new generation of multi-lineage organoids is being developed to meet the growing demand for understanding the interactions among multiple subregions or multiple cell types in organs.

Achievements have made in the field of multi-lineage brain organoids. Multiple groups have fused hPSC-derived organoids of different brain regions and found that the fused organoids can model human brain development, interneuron migration, neuronal projection and neurodevelopment disorders with enhanced success via complex cellular and regional interactions.^35,42,71,175,176^ Such multiregion organoids combining multiple cell lineages in three dimensions are also called “assembloids”.^177^

Some studies have also combined neural cells with other lineages of cells. For example, coculture with endothelial cells has been found to recapitulate vascularization and even BBB formation in brain organoids.^178,179^ Microglia-like cells derived from hPSCs have been integrated into cortical organoids to study neural–glial interactions in Alzheimer’s disease.^180^ Additionally, assemblies of cortical, spinal, and muscle organoids can resemble the corticomotor circuit in vitro long term and be used to model cortical control of muscle contraction.^181^ Faustino Martins et al. demonstrated that human neuromuscular organoids were derived from hPSC, which could reconstruct neuromuscular junctions and be applied to investigate the pathogenesis of the neuromuscular disease.^182^ In addition, intestinal organoids derived from hiPSCs can be combined with neural crest cells to reproduce the developmental stage of the intestinal nervous system and study (gastrointestinal) GI motility disorders.^183^ In a similar way, glioblastoma cells from patients have been combined with brain organoids to study tumorigenesis in the nervous system.^184^

Assembloid-based multi-lineage studies have also been reported on other organs. For instance, Hiroyuki Koike et al. demonstrated that foregut- and hindgut-like tissues developed from hPSCs exhibit dynamic morphogenesis and continuous patterning of hepatic, biliary, and pancreatic structures.^185^ Moreover, the liver organoids established by Ramli et al. contained hepatocytes and cholangiocytes with a functional bile canalicular system, and this multi-lineage culture could be used to model complex liver disease.^113^ A recent study recapitulated human cardio-pulmonary co-development using simultaneous multilineage differentiation of hiPSCs, which would potentially offer a system to investigate human cardio-pulmonary interaction and tissue boundary formation during embryonic development.^186^

It is undeniable that it is still difficult to truly replicate the interplay among organ-organ and even cell-cell in the real human body. However, with the continuous progress of organoid techniques, multi-lineage model systems will provide new tools to reveal the complexity and display the interactions of multiple regions or cells. In this way, the multi-lineage organoids may enable more specific approaches to fight disease and discover potential targets for therapy.

## Applications of organoid technology

**Fig. 5:**
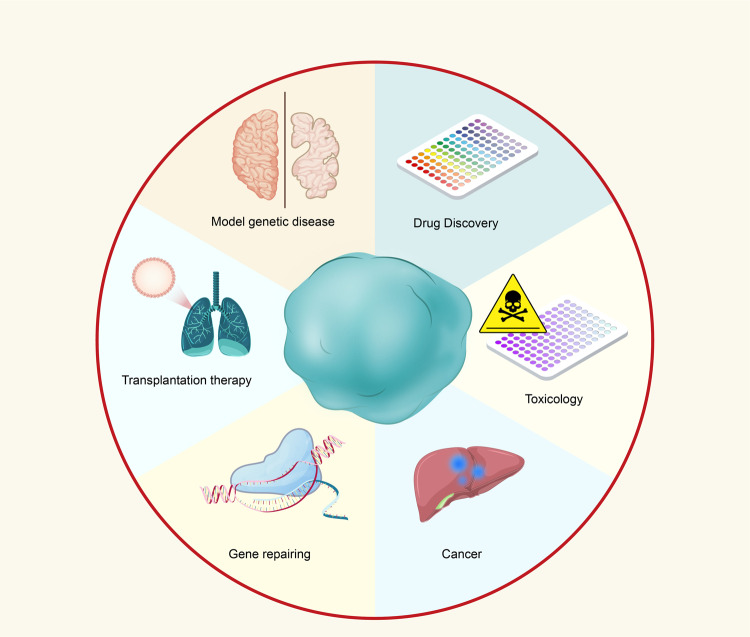
Various applications of organoid technology. Schematic depiction of various applications of organoid technology based on the studies mentioned above. Organoid technology provides ideal models mimicking human genetic diseases caused by induced mutations. By using gene editing techniques such as CRISPR/Cas9, researchers could investigate some diseases associated with genetic defects. Organoid technology has also provided a potential tool for high-throughput drug discovery and enables accurate toxicity testing and preclinical studies. Recent advances in cancer research within organoids are opening the way for promising organoid transplantation therapy in the future

In vitro organoids recapitulate the principles of organ biology and offer simplified and easily accessible cellular model systems that mimic specific aspects of the 3D architecture, composition of cell types, and human organ functions. Organoid technology may fill a need in medical research and holds promise for a wide range of translational applications (Fig. 5).

## Modeling genetic disease

Two primary applications of human organoids are modeling of human genetic diseases and the development of new therapeutic strategies. Hans Clevers et al. established intestinal crypt organoids for the first time with adult intestinal stem cells.^12^ Then, gut organoids were applied to study cystic fibrosis (CF), a genetic disease caused by mutations of the CF transmembrane conductance regulator (CFTR). A quick and robust assay for the quantification of CFTR function was thus established that can accelerate diagnosis, functional research, drug screening, and personalized medicine strategies in CF studies.^187,188^ After gut organoids were successfully constructed, increasing numbers of different kinds of organoids were established and used for genetic disease modeling. Menendez et al. generated kidney organoids generated from a patient with polycystic kidney disease, which established a foundation for studying other genetic kidney disorders.^189^ Recently, kidney organoids derived from hiPSCs with GLA-mutation were used to study the mechanisms and to develop new treatments for Fabry disease.^190^ Retinal genetic diseases have also been modeled with retinal organoids.^191,192^ Similarly, NKX2-5- and HAND1-knockout (KO) cardiac organoids have been applied to model hypoplastic left heart syndrome, the most severe congenital defect in humans.^169^ In particular, brain organoids have been widely used for research on various neurodevelopmental genetic disorders. For instance, microcephaly, a disorder associated with CDK5RAP2 mutations that is difficult to recapitulate in mice, has been modeled with patient-specific iPSC-derived cerebral organoids.^13^ Other neurological disorders, including Down syndrome, Alzheimer’s disease, and PD, have also been modeled with brain organoids.^34,42,76,193–199^

## Drug discovery and toxicity assessment

Current medical discoveries of human diseases usually confront with limitations, such as individual diversities in patient, unpredictable outcomes, and time-consuming drug testing.^200^ 3D organoids based on a specific disease have shown great potential in drug screening.^8,15,201^ Organoids derived from iPSCs of patients with primary cancers, infectious diseases, or developmental diseases show similar clinical phenotypes and may be potential platforms for new drug testing. Broutier et al. identified an ERK inhibitor as a potential therapeutic treatment with human primary cancer organoids in 2017.^18^ In the same year, Crespo et al. developed colonic organoids from patients with familial adenomatous polyposis coli (FAP) that incorporated mutations in a negative regulator of the WNT pathway called the adenomatous polyposis coli (APC) gene.^202^ They also screened two compounds that were effective in rescuing the overproliferation of patients’ organoids, which could also affect wild-type organoids. Except for cancer organoids, brain organoids have been used for drug discovery. Cortical organoids infected with ZIKV display phenotypes similar to those of congenital Zika syndrome, including a thinner neuronal layer and dilation of the ventricular lumen.^32,203–205^ In several studies, virus-infected brain organoids have been applied to provide platforms for validating antiviral drug candidates, such as duramycin, ivermectin, and azithromycin.^14,206^ High-throughput drug screening is usually performed on intestinal organoids. For example, Kenji Kozuka et al. cultured mini colonic organoids in 96-well plates and performed a successful screen of approximately 2000 compounds for potential drugs.^207^ Another case is an approach related to the development of anti-diarrheal drugs and was proposed by Onur Cil’s group.^208^

Toxicity assessment and preclinical studies of drugs at this stage have certain limitations, and the toxic reactions of many drugs are only gradually discovered in clinical studies or at the launch stage. Organoids could be used to test experimental drug reactions because of the similarity of their reactions to physiological tissue reactions.^209^ Therefore, it is promising to establish a long-term toxicity screening model with human physiological characteristics by using organoids.

Organoids can be used to assess drug toxicity, such as side effects on the liver,^210^ heart^211^, and kidneys.^93^ Skardal et al.^212^ developed an organoid system consisting of heart, lung, and liver organoids within a single recirculating perfusion system in a common medium system with biosensing capabilities that could be used to assess the pharmacological effects and toxic responses of drugs on whole organs.

## Cancer

Organoids have deepened our knowledge of human cancers. A major attribute of organoids that makes it significant in cancer research is the organoids derived from patient could mimic the tumor characteristics. Traditionally, animal cancer models, human cancer cell lines or primary patient-derived tumor xenografts (PDXs) have become important tools for cancer research. Recently developed AdSC-derived organoid technologies aid transformed cancer tissues cultured in vitro as cancer organoids. Colorectal cancer organoids derived from patients were first established in 2011,^213^ and other varieties of cancer organoids have since emerged.^214–220^ Unsurprisingly, these various cancer organoid models show an excellent ability to phenocopy human tumors. Using these models, not only drug screening^15,221^ but also basic research can be conducted. For instance, a coculture system has been used to study the relationships of infectious agents such as *H. pylori* with gastric cancer, and the affected stomach organoids have exhibited pathological phenomena similar to those of cancer.^140,222^ The use of organoid technology is thus a promising approach for better understanding cancer and translating preclinical drugs to the clinic.

## Personalized medicine

For many cancers, traditional cell culture methodologies are unable to sufficiently model the biology of native tumors, thus contributing to the high failure rates of preclinical compounds in clinical trials.^223,224^ Given the superiority of in vitro patient-derived organoids (PDOs) in rapid growth and stable differentiation and the capacity of PDOs to capture patient and tumor type diversity, organoids could potentially be used to identify effective therapeutic strategies for a particular patient in so-called personalized therapy. In recent years, massive efforts have been made to successfully establish biobanks of organoids generated from various tumors, including prostate,^225^ lung,^226^ colorectal,^227^ liver,^18^ pancreatic,^228^ and gastric^229^ tumors. The most suitable drug for patients can be quickly detected through organoid drug sensitivity testing; thus, the most effective drug treatment plan can be formulated, which may minimize the drug side effects and reduce tumor recurrence. This strategy may enable the ideal treatment schemes to be selected. A prime example is CF treatment, which is a life-shortening disease caused by CFTR gene mutation that brings particularly severe damage to the pulmonary, digestive, and urinary systems.^230,231^ Berkers et al. clarified the correlation between the response of forskolin-induced swelling (FIS) of a CF patient-derived rectal organoid model and the in vivo therapeutic responses of individual CF patients with a variety of CFTR mutations who have received treatment with several CFTR-modulating drugs, and this is the first clinical application of organoids to guide individualized medication.^232^ This study raised interest in using the FIS assay as a prospective biomarker to quantitate individual CFTR modulator clinical responses.^232^ Several studies also have already investigated that rectal cancer organoids can be used to predict responses in the clinical radiotherapy.^233–235^ Yao et al. established a living organoid biobank from individuals with locally advanced rectal cancer (LARC) and showed that the effects of neoadjuvant chemoradiotherapy (nCRT) on organoids in vitro correspond significantly to the effects on patient tumors, indicating that PDOs could predict chemoradiation responses of LARC patients in the clinic.^234^ Similarly, Ganesh et al. observed a high correlation between the responses of LARC PDOs and the responses of patients’ tumors to radiation or chemotherapy.^235^ Screen of drug candidates is another important clinical application of PDOs. Recently, a living biobank was established from 30 patients with pancreatic ductal adenocarcinoma (PDAC), with the aim to high-throughput drug screening for 76 therapeutic agents. The results revealed in vitro sensitivity to these agents that are currently not exploited in the clinic and showed that the PRMT5 inhibitor EZP015556 can be effective in both methylthioadenosine phosphorylase (MTAP)-negative PDOs and a subset of MTAP-positive PDOs, demonstrating the importance of personalized approaches for cancer therapy.^228^ Clinical trials with this inhibitor are now ongoing (NCT03573310, NCT02783300, and NCT03614728).^228^ Collectively, these findings suggest that PDOs have the potential to be powerful, attractive tools for modeling disease and screening personalized drug, opening a novel avenue to precision medicine.

## Gene repair and transplantation therapy

Gene repair treats various diseases, such as cancer, diabetes, heart disease, and acquired immunodeficiency syndrome (AIDS), by altering the target genes inside the body’s cells. Since genetically modified Lgr5^+^ stem cell-derived organoids have been successfully generated and transplanted into damaged tissues,^236,237^ gene repair has become increasingly promising. In recent years, the CRISPR/Cas9 technologies have been applied for gene repair because of its high gene editing efficiency.^7,238^ Schwank et al. found that CFTR function was corrected after the human CFTR mutation F508del was corrected with a CRISPR/Cas9-mediated homology-dependent repair system and demonstrated the functionality of gene repair, providing a potential gene therapy for CF patients.^7^ Notably, this therapy has achieved the first successful CRISPR/Cas9 gene-editing case in human-derived organoids.^239^ Since this CRISPR/Cas9-mediated homology-dependent repair system may give rise to potentially harmful off-target double-strand breaks, a more advanced technology named CRISPR-based adenine editing (ABE) has been developed. This method enables on-target base editing, including accurate enzymatic conversion of A-T base pairs into G-C base pairs, which helps repair the function impaired by CFTR mutation.^238^ Considering this progress, gene editing can undoubtedly repair genetic mutations in PDOs, rapidly propelling gene repair into the clinical stage.^240^

Organ transplantation is essential resources required for treating patients with organ failure. However, many challenges remain, especially severe graft rejection and an increasing shortage of organ donors. Infinitely available in vitro-cultured organoids have become potential donors, providing a promising platform for autologous transplantation therapy.

To make organoid transplantation therapy a reality, researchers have made many efforts to verify its feasibility. For instance, Watson et al. transplanted an intestinal organoid derived from hPSCs into a host mouse and found that the transplanted organoid showed marked expansion and maturation.^241^ McLelland et al. demonstrated that transplanted sheets of retinal organoids could form synaptic connections and extensive projections in host rats with retinal disorders.^242^ Chen et al. observed that lung bud organoids showed branching morphology and proximodistal specification similar to those observed in vivo at 5 months after ectopic transplantation.^120^ Wang et al. developed cerebral organoids and then implanted them into a stroke rat model.^243^ They observed reduced brain infarct volumes and neurological motor function improvement after transplantation. Notably, our group has confirmed that human cerebral organoids can functionally integrate into neural circuits and establish subcortical projections within host mice.^244^ Many other studies on organoid transplantation have been performed, providing an important theoretical basis for the exploration of transplantation therapy.^245–248^

Although transplanted organoids are relatively immature compared with the host’s natural organs because of their incomplete functional maturation and possible heterotypic cell interactions, the therapeutic use of organoids will still be a meaningful therapeutic alternative to organ transplantation.

## Conclusion and perspective

Human organoids hold great promise for applications ranging from basic research to biomedicine. Disease modeling using organoids has naturally been associated with preclinical testing in the drug development stage, and high-throughput drug screening using organoids have been extensively utilized in the field of cancer research.^228,249,250^ In addition, cancer organoids may be potential tools for identifying effective drugs in cancer patients.^228,251^ For instance, colorectal cancer patients with different genotypes have been shown to exhibit different sensitivities to EZH2 inhibitors.^251^ Organoid systems might also be applicable for drug toxicity and safety testing because of their effectiveness.^252^ Moreover, 3D organoid technologies have provided attractive platforms for studying host–pathogen interactions in various human infectious diseases, including the infection of protozoan parasites, bacteria, and viruses. For example, a recent study reported that cerebral organoids could be used to investigate the underlying mechanisms of ZIKV-caused microcephaly, and ZIKV-infected organoids were found to be smaller than controls, in line with the symptoms observed in patients.^32,204^ Another inspiring example involves the recent necessity to develop organoid models for the emerging SARS-CoV-2 pandemic. The current coronavirus disease 2019 (COVID-19) pandemic caused by SARS-CoV-2 is a potentially fatal disease with a higher death rate in humans than common influenza. Developing vaccines and therapeutics against COVID-19 is an urgent matter, and human organoids have provided valuable study tools for pathomechanistic research on SARS-CoV-2. Several studies showed that SARS-CoV-2 infected AdSC-derived organoids generated from human airway^253^ and gut^254^ as well as in PSC-derived blood vessel,^255^ brain,^256^ and kidney^257^ organoids. Organoids are also emerging as potential sources of transplantable tissues and functional cell types for cell replacement therapies in regenerative medicine. Many successful attempts have been made to transplant various kinds of organoids into hosts, including retinal, colonic, intestinal, liver, and kidney organoids; these organoids have been shown to effectively integrate with the host tissue or be able to reconstruct the organ function to a certain degree.^22,237,245,258^ Autologous replacement therapy would also allow the combined use of genetic correction strategies for disease-related mutations.

**Fig. 6:**
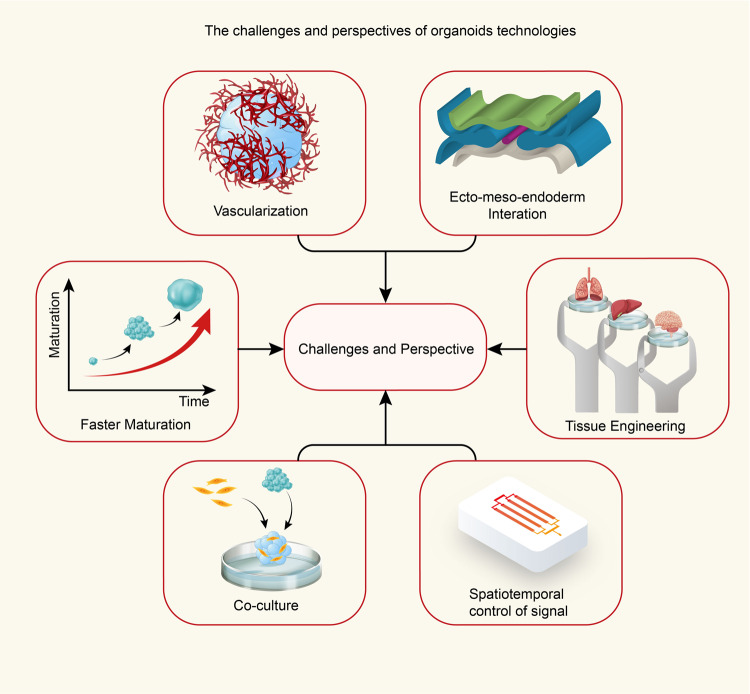
Challenges and perspectives of organoid technologies. View of the challenges and perspectives of organoid technologies. Specifically, the sizes of the organoids developed to date have been limited due to the lack of vascular structures, but implementation of vascularization via coculture with endothelial cells could provide a near-physiological way to increase nutrient exchange. Slow maturation is another crucial factor limiting the disease modeling of later developmental stages, but pretreating organoids with small molecule compounds may be a solution to this hurdle. Moreover, establishment of ecto-, meso-, and endoderm tissue interactions during development may be a promising approach to improve recapitulation of the typical organ system interactions observed in vivo. Tissue engineering could control over the materials and the precise apportion of different cell types through kinds of biofabrication techniques, it is also a promising approach to using microfluidic platform in composite organoids. Coculture systems could offer the advantages of 3D organoid model systems to study functional and/or temporal and spatiotemporal interactions among diverse cell populations. Following the principles of organogenesis during the embryonic developmental stage, the regulation of cell differentiation in space and time can be directly controlled in vitro via implementation of concentration gradients and signaling centers of shape-guided morphogens

Although the 3D organoid field is moving ahead and offering innovative approaches for biomedical research and clinical translational research, there are still several hurdles that need to be addressed (Fig. 6).

First, human organoid cultures depend on mouse-derived ECM substitutes, such as Matrigel and basement membrane extracts. These extracts showed component variability due to batch production, which affects the replicability of experimental results in biologic research. In addition, they may hinder direct clinical transplantation due to their unknown pathogens and the potential immune responses they elicit. This shortcoming may be able to be solved via culture with clinical-grade collagen.^237^ Bioengineering strategies will also provide new directions to improve the methodology.^259^

Another factor limiting the use of current organoid culture methods in future regenerative medicine applications is the inability of these approaches to model multiorgan pathologies. Coculture approaches could partially address this issue. A recent related attempt has been reported involving a combination of hPSC-derived intestinal organoids and neural crest cells.^183^ Moreover, recent studies have devised strategies to create organ-on-a-chip technologies in order to generate 3D systems that enable connection and communication among multiple preformed ‘organs’.^260^

Tissue maturation is a third crucial factor that restricts organoid technology from reaching its full potential for preclinical and even clinical applications. For example, organoid culture systems replicate the fetal brain development stage to a large extent, which makes it difficult to model neurological disorders that affect the adult brain, such as Alzheimer’s disease. To overcome these barriers, many protocols have been optimized to include pretreatment of organoids with small-molecule compounds such as BDNF^261^ in order to accelerate maturation. Oxygenation and nutrient diffusion are two other key factors in the process of maturation. Long-term culture of organoids does not expand along with time, but rather leads to the immediate apoptosis/necrosis to form a cavity in the core of organoid, due to anoxia.^262–264^ This is observed relatively frequently in cerebral organoid culture systems. It has been suggested that when the diameter of cerebral organoids is up to approximately 500 µm, they might begin to exhibit metabolic cell stress, thus leading to a decreased richness of cell subtypes in comparison with aborted fetal brain tissue.^265,266^ To resolve this issue, organoids were initially grown in a spinning bioreactor or using a shaker. Other attempts have been made to devise a variety of technical strategies for vascularization. For instance, whole-brain organoids have been re-embedded in Matrigel containing iPSC-derived endothelial cells to form vascularized organoids, which further promotes organoid growth and supplies nutrients.^36^ A recent study has reported that transplantation of organoids into animals results in vascularization from the host brain; the resulting vessels can transport oxygen and nutrients to support improved survival of neurons within the graft and progressive maturation of brain organoids.^267^ Furthermore, the incorporation of biomaterials, nanotechnology, bioengineering, and other advanced protocols may enable the enlargement and long-term culture of human organoids in the future.^268^

The limited reproducibility that processes a major hurdle to generating higher-grade organoids and gaining control of their functionalities is another confounding issue. The essential factors affecting the reproducibility of organoids are basically containing batch to batch variation, organoid production scalability, cellular composition, and architecture of organoids. Krefft and his team have established a forebrain organoid approach that reduces the heterogeneity by adding the patterning factors based on the self-organizing ability of iPSCs.^269^ Tackled these challenges, it is foreseeable that a relatively accurate, reproducible 3D model may emerge, realizing the transition of organoid technology from scientific research to clinical practice and accelerating larger-scale organoid manufacturing for drug screening.

Notably, unlike PSC-derived organoids, AdSC-derived organoids only represent the epithelial parts of organs, thus summarizing the typical structure and function of the primitive epithelium; in contrast, the compartments of stroma, nerves, immune cells, and vasculature are absent.^28^ Therefore, ASC-derived organoids exhibit lower structural complexity than PSC-derived organoids, but are closer to adult tissue.

Overall, emerging organoid technologies have already been useful for biomedical research, for drug screening in individualized medicine and, in combination with genome-editing technology, for gene therapy. The broader applications of organoids are in the beginning stage of exploration. Extensive research will enable 3D organoid systems to replenish existing model systems, which could strengthen basic and clinic studies in the future.
